# Estimating the cost-effectiveness of lifestyle intervention programmes to prevent diabetes based on an example from Germany: Markov modelling

**DOI:** 10.1186/1478-7547-9-17

**Published:** 2011-11-18

**Authors:** Anne Neumann, Peter Schwarz, Lars Lindholm

**Affiliations:** 1 Epidemiology and Global Health, Department of Public Health and Clinical Medicine, Umeå University, Umeå, Sweden; 2 Cancer Epidemiology, University Hospital, Technical University of Dresden, Dresden, Germany; 3 Department of Internal Medicine, Carl Gustav Carus Medical School, University of Technology Dresden, Germany

**Keywords:** Diabetes mellitus, health care costs, health care economics and organisations, primary prevention, life style, early intervention, decision making

## Abstract

**Background:**

Type 2 diabetes mellitus (T2D) poses a large worldwide burden for health care systems. One possible tool to decrease this burden is primary prevention. As it is unethical to wait until perfect data are available to conclude whether T2D primary prevention intervention programmes are cost-effective, we need a model that simulates the effect of prevention initiatives. Thus, the aim of this study is to investigate the long-term cost-effectiveness of lifestyle intervention programmes for the prevention of T2D using a Markov model. As decision makers often face difficulties in applying health economic results, we visualise our results with health economic tools.

**Methods:**

We use four-state Markov modelling with a probabilistic cohort analysis to calculate the cost per quality-adjusted life year (QALY) gained. A one-year cycle length and a lifetime time horizon are applied. Best available evidence supplies the model with data on transition probabilities between glycaemic states, mortality risks, utility weights, and disease costs. The costs are calculated from a societal perspective. A 3% discount rate is used for costs and QALYs. Cost-effectiveness acceptability curves are presented to assist decision makers.

**Results:**

The model indicates that diabetes prevention interventions have the potential to be cost-effective, but the outcome reveals a high level of uncertainty. Incremental cost-effectiveness ratios (ICERs) were negative for the intervention, ie, the intervention leads to a cost reduction for men and women aged 30 or 50 years at initiation of the intervention. For men and women aged 70 at initiation of the intervention, the ICER was EUR27,546/QALY gained and EUR19,433/QALY gained, respectively. In all cases, the QALYs gained were low. Cost-effectiveness acceptability curves show that the higher the willingness-to-pay threshold value, the higher the probability that the intervention is cost-effective. Nonetheless, all curves are flat. The threshold value of EUR50,000/QALY gained has a 30-55% probability that the intervention is cost-effective.

**Conclusions:**

Lifestyle interventions for primary prevention of type 2 diabetes are cost-saving for men and women aged 30 or 50 years at the start of the intervention, and cost-effective for men and women aged 70 years. However, there is a high degree of uncertainty around the ICERs. With the conservative approach adopted for this model, the long-term effectiveness of the intervention could be underestimated.

## Background

Type 2 diabetes mellitus (T2D) poses a large worldwide burden for health care systems as its prevalence is high and treatment expensive. The International Diabetes Federation (IDF) estimated that about 258 million people around the world suffered from diabetes mellitus in 2010, and the majority have T2D [1]. IDF estimates that health care expenditures account for 11.6% of the world's total health care expenditures [1]. Health care costs related to T2D increases with T2D complications [2]. Furthermore, T2D is commonly detected late in the course of disease. The time between T2D onset and diagnosis can be up to ten years [3]. In consequence, effective intervention programmes that address major risk factors for T2D development, ie, lack of physical activity and unhealthy diet, could lead to a decreased T2D prevalence, reduction in costs due to treatment and inability to work, and shorten the time between onset and diagnosis of disease which may reduce complication rates.

Lifestyle interventions for prevention of T2D will need to be evaluated. Examples of such interventions are the Prevention of Diabetes Self-Management Program (PREDIAS) [4] and the Saxon Diabetes Prevention Programme (SDPP) which are described in more detail below. Both target individuals at high risk of developing T2D. The lifestyle interventions are comprised of motivation analysis, exercise programmes and dietary counseling.

Medicine and public health expect high value from primary prevention of T2D, but not much is known about its long-term cost-effectiveness. Saha et al [5] recently reviewed economic evaluations of lifestyle interventions for primary and secondary prevention of diabetes and cardiovascular disease. They identified a range of decision analytic models. However, only three models include the state of normal glucose tolerance and this is a necessary state to accurately analyse the impact of such interventions [6-8].

Decision modelling is an effective and relatively inexpensive technique for extrapolating available, evidence-based information on the epidemiology, pathogenesis and intervention results of a disease in order to estimate costs and effectiveness over a longer time span [9]. Sculpher, Fenwick and Claxton [10] describe the role of cost-effectiveness models as to "identify optimum treatment decisions in the context of uncertainty about future states of the world."

Public health pioneers also discuss uncertainty. In 1992, the famous epidemiologist Geoffrey Rose wrote "It needs to be better understood by the public, by policy-makers, and by medical scientists alike that we can never be certain of anything. Certainty is not a prerequisite for action." [11].

Uncertainty is embedded in every result from every study. The greater the complexity of the disease, the higher the uncertainty in the analysis. Decision-making under uncertainty is always a balancing act between being timely and having data available. If ideal data (eg, changes in mortality and morbidity) are not available, we must use the best available evidence to estimate meaningful outcomes.

This analysis uses decision modelling to further knowledge on primary prevention of T2D. We evaluate the cost-effectiveness of lifestyle intervention programmes to prevent T2D. We calculate costs from a societal perspective, ie, costs from different perspectives are considered. The goal of this study is to provide public health decision-makers with information that can be used to enhance decision-making about primary prevention of diabetes.

## Methods

A model evaluating a hypothetical intervention programme is used. The targetted programmes are described below. Two examples for those programmes in Germany are PREDIAS and SDPP. Lifestyle interventions for prevention of T2D target individuals at high risk of developing T2D. The lifestyle interventions are comprised of motivation analysis, exercise programmes and dietary counselling. Each intervention is proven to be effective and are the cornerstones for successful implementation of a lifestyle intervention programme [12-14]. The programmes typically have prevention managers who organise group-based interventions and are the direct contact persons for the participants.

The interventions consist of three steps. First, high-risk individuals are identified with an easy, fast and low-cost screening tool. An example is the Finnish Type 2 Diabetes Risk Score (the FINDRISK). Screening tools are distributed through community promotion and advertising, eg, informational leaflets at health insurance companies, primary care physician offices, and health fairs [15]. FINDRISK is a self-administered questionnaire using eight simple questions to estimate risk of developing T2D in the next 10 years. Questions ask for information such as body mass index (BMI), age, and waist circumference. Individuals with a FINDRISK final sum score below 11 are considered low risk, receive basic information about healthy lifestyle, and are reminded to complete the FINDRISK questionnaire again in the future. Those with a final sum score of 11-20 are considered high-risk and invited to join a lifestyle intervention course. Individuals with a final sum score of 21 or greater receive a recommendation to visit their general practitioner for T2D testing. If they do not have T2D, they are invited to join the intervention programme. Second, people are encouraged to participate in a structured programme aimed at changing lifestyle. Skilled staff certified in T2D prevention, eg, experts in nutrition and physical exercise and with additional education in diabetes prevention, hold courses once a week for eight weeks. The course consists of lectures about the physiology of the body, healthy eating, exercise and motivation that are given to groups of approximately ten participants. Third, participants are offered follow-up mentoring, which can continue as long as they wish [16]. The prevention manager coodinating the course is in continuous contact with the participants and reminds them to reach and maintain their goals. The participant has regular email and telephone support and receives a monthly newsletter and quarterly journal that provide information about aspects of a healthy lifestyle. At regular intervals, the prevention manager collects objective measurements on participants, ie, blood pressure, weight, and waist and hip circumferences. The lifestyle intervention can raise awareness through personal contact, the a website, journals and newspapers, personal invitation letters, and health fairs. If an individual is interested, he or she receives a folder with further information.

Markov modelling with a probabilistic cohort analysis was used to calculate the cost per QALY gained of the above described lifestyle intervention in comparison to no intervention.

Decision modelling for health economic evaluation in general and further details specific to this model are explained elsewhere [17,18].

**Figure 1:**
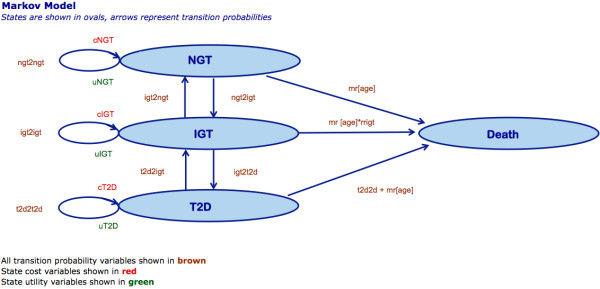
**Markov Model Schematic**.

Four health states were modelled in a cohort simulation. An individual in this simulation could have normal glucose tolerance (NGT), impaired glucose tolerance (IGT), diagnosed type 2 diabetes mellitus (T2D), or be dead. Figure 1 displays a schematic of the model. The simulations were conducted with Microsoft^® ^Office Excel^® ^2004 for Mac.

Each state portrays aggregate values while an individual in each state can have different complications. We applied a one-year cycle length and a lifetime time horizon. The lifetime time horizon indicates that individuals will be followed life-long for all possible outcomes that might arise. The model assumes participants only participate in the intervention for five years and the effectiveness of the intervention lasts for six years, with a linear decrease in effectiveness during the six years with no effectiveness difference in the seventh year. The Finnish DPS found that after discontinuation of active counselling, the lifestyle intervention group had a lower incidence of T2D after seven years compared to the no-intervention group [19]. The current study selects input model parameters from different studies as no single source could be identified for all of the necessary data. Cost-effectiveness acceptability curves are used to illustrate final results to help decision-makers. In the following, input parameters are discussed in more detail.

### Transition probabilities

Table 1 summarises the one-year transition probabilities chosen for the model.

**Table 1:** Transition matrix between health states

Transition Matrix Intervention (No-intervention) *			
**To** **From**	**NGT**	**IGT**	**T2D**

**NGT**	0.848 (0.837)	0.152 (0.163)	-

**IGT**	0.177 (0.162)	0.794 (0.775)	0.03 (0.06)

**T2D**	-	0.005 (0.005)	0.995 (0.995)

* Please refer to figure 1 for a schematic overview

Most studies of T2D development focus on the transition from IGT to T2D. However, it is important to model from the time when a person has NGT as prevention ideally starts before development of IGT and its higher risk. Transition probabilities between NGT and IGT were taken from a Canadian study [6]. Data on primary prevention of IGT is sparse and most models start with the state of IGT. The study by Caro et al [6] is unique in its calculation of one-year transition probabilities between NGT and IGT. The one-year transition probability for no intervention was 16.3% and 15.2% for the lifestyle intervention [6]. The one-year probability of converting from IGT to NGT was 16.2% for no intervention and 17.7% for the intervention [6]. The Finnish DPS evaluated the risk of T2D development in persons with IGT. In the DPS, transition from IGT to T2D was 6% for no intervention and 3% for the intervention [20].

We used a probability of moving from T2D to IGT of 0.5% for both the no-intervention and intervention groups. These assumptions are based on the knowledge that this transition exists but seldom occurs. The transition probability in the intervention group is likely higher than in the no-intervention group, but we took a conservative approach and did not assign a difference between the groups.

The prevalence of IGT among the general German population is used as the base for the model. A population-based study in the region of Augsburg, Germany, found that approximately 16% of healthy individuals developed IGT [21]. Thus, we assume that at the beginning of the model 16% of individuals have IGT, 84% has NGT and no one has T2D.

#### Duration of expected effects

The model takes a conservative view by setting the effectiveness of the intervention to last only for six years, with a linear decrease in effectiveness during the intervention. Thus, from year 7 and onwards the same transition probabilities are applied for both intervention and no-intervention groups. Seven-year effectiveness was selected because the DPS showed a 36% reduction in relative risk of progression from IGT to T2D between the intervention and the control group after seven years [19]. Furthermore, the Chinese Da Qing Diabetes Prevention Study estimated that the lifestyle intervention group had a 51% lower incidence of diabetes during the active intervention period and a 43% lower incidence over the whole 20 year period compared with control participants [22]. Our model includes people with NGT and IGT whereas the Da Qing Diabetes Prevention Study and the DPS only included people with IGT in their studies. We chose to be more conservative than either of these studies and assumed a seven-year effectiveness to be sufficiently conservative.

### Mortality

Life tables provide the mortality rates for different ages and sexes. Eight different mortality categories, by age and sex, are established: less than 35, 35-64, 65-74, and 75 years and over for men and women. The groups are estimated to give appropriate categories that are relatively heterogeneous between groups and homogenous with groups in regard to mortality.

#### Mr[age]

Mr[age] symbolises the probability of dying in a certain age and sex group when a person does not have T2D. Mortality statistics were obtained from the Statistical Office of the Federal State of Saxony [23]. The number of those dying due to T2D (t2d2d) was subtracted from the total number of deaths in Saxony during 2006.

#### T2d2d

T2d2d is the transition probability of a person with T2D dying from T2D. In most statistical records, this is underestimated as many death certificates do not record T2D as the underlying cause of death [24]. We used the percentage of all-cause deaths attributable to diabetes in the "Europe region with very low child and adult mortality" from the study by Roglic et al [25]. The all-cause death figures were taken from mr[age] as described above. T2d2 was age and sex dependent.

### Health care cost in different states

German estimates for health care costs in different states were used as the cost estimates of the lifestyle programme derived from a German setting.

#### cNGT

cNGT is the estimation of health care costs for someone with NGT. The CODE-2 study calculated an average annual direct health care cost of EUR1,372 for a person with NGT in Germany [2]. This is the average cost for someone without diabetes who is insured by the German statutory health insurance. As this cost was collected in 1997, we calculated a 2007 equivalent based on a formula from the Federal Statistics Office in Germany which was adapted for health care costs [26]. The NGT cost of EUR1,744.21 is adjusted for the difference in purchasing power.

#### cIGT

cIGT is the estimation of health care costs for someone with IGT. Unlike cNGT, no comparable cost estimation could be located for the annual cost for IGT. As the three main cost states need to be comparable, the ratio used by Palmer et al [27] that consists of 46% of diabetes costs, was adapted to the CODE-2 costs. The annual cost for IGT is estimated at EUR2,696.48, ie, 46% of EUR5,861.92 (see next paragraph).

#### cT2D

cT2D is the estimation of health care costs for someone with T2D. According to the Code-2 study, the average annual cost for a diabetic patient in Germany is EUR4,611 [2]. Adjusting for inflation, the cost for T2D is EUR5,861.92 [26].

#### Cost of the Intervention Course and Follow-up Mentoring

We analysed the course of the SDPP lifestyle intervention. All costs are expressed in Euros and prices are adjusted to 2007 values. The cost of the intervention consists of costs for screening (ie, information folders), the course and follow-up mentoring. Other costs such as transportation (eg, cost of driving to educational places) are also considered.

The cost of the intervention is calculated as follows:

Screening costs consider direct costs associated with preparation and mailing the information folder to interested individuals before they join the programme. Course costs are comprised of the telephone hotline, email mentoring, newsletters, journals, collection of medical data, postage, and the time of the prevention manager. The prevention manager's course time is eight hours plus one hour for each course participant. Costs for follow-up mentoring include the telephone hotline, email mentoring, monthly newsletters, quarterly journals, biannual collection of medical data, postage and the prevention manager's time. Prevention manager follow-up time includes four hours for the four events plus one hour preparation time for each, 30 minutes per month counselling per participant, and 30 minutes per participant biannually for collection of medical data.

Participant driving costs to educational locations were estimated by using an average of 5 km driving distance multiplied by the general mileage allowance for Germany.

Table 2 gives an overview of the intervention costs. The first year costs are approximately EUR390 and the second and subsequent years are approximately EUR190 per year.

**Table 2:** Assumed intervention costs

Screening (per participant)	EUR113.32
	

Mailing informational folders	EUR 113.32

*52 TN/244 folders = 21.3%, one folder = EUR24.15*	

	

**Course (per person)**	EUR109.16

Phone hotline	EUR3.80

Email mentoring	EUR3.80

Newsletter	(8 × EUR1.75) EUR14.00

Journal	(2 × EUR1.68) EUR3.36

Collection of medical data	(2 × EUR0.15) EUR0.30

Postage	(2 × EUR0.35) EUR0.70

Prevention managers (contact hours + preparation)	(26 h × EUR32/10 persons) EUR83.20

	

**Follow-up mentoring** **(per person per year)**	EUR183.93

Phone hotline	(2 × EUR3.80) EUR7.60

Email mentoring	(2 × EUR3.80) EUR7.60

Newsletter	(12 × EUR0.90) EUR10.80

Journal	(4 × EUR1.49) EUR5.96

Collection of medical data	(2 × EUR0.15) EUR0.30

Postage	(4 × EUR0.35) EUR1.40

Events, 10 person group, 2 times per year	(2 × EUR45/10 persons) EUR18.00

Prevention managers (contact hours + preparation)	(41.33 × EUR32/10 persons) EUR132.27

	

**Other costs to be considered**	

Driving to educational locations, one round-trip	(2 × 5 km × EUR0.30/km) EUR3.00

**Summary**	

**Screening**	**EUR113.32**

**Intervention, including transportation costs**	**EUR133.16**

**Follow-up, including transportation costs**	**EUR143.95**

**Total cost of year 1**	**EUR390.43**

**Total cost of follow-up for year 2 and each following year**	**EUR189.93**

The participants are assumed to participate in the intervention for five years. Thus, the costs for the intervention only occur for five years.

### Utility weights

A study by Kontodinopoulos et al provides health utility values for the general Greek population and for a Greek population with T2D [28]. Table 3 shows an overview of the utility values in that study (mean and standard errors). The standard errors were calculated on the basis of reported standard deviations [29].

**Table 3:** Overview of utilities from a Greek population [28]

	Men (SE)	Women (SE)
**health utility value for NGT (uNGT)**	0.772 (0.004)	0.747 (0.004)

**health utility value for IGT (uIGT)**	0.764 (0.006)	0.740 (0.006)

**health utility value for T2D (uT2D)**	0.724 (0.010)	0.701 (0.010)

#### uNGT

We assumed that the general Greek population in the study of Kontodinopoulos et al [28] is equivalent to the state of NGT. This assumption probably underestimates the utility value for NGT (uNGT) as people in the general population might have undiagnosed IGT. The utility value was 0.772 (SE: 0.004) for men and 0.747 (SE: 0.004) for women [28].

#### uIGT

The utility value for IGT (uIGT) was estimated as a 1% decrease from uNGT. Therefore, we used a utility value of 0.764 (SE: 0.006) for men and 0.740 (SE: 0.006) for women.

#### uT2D

The utility value for T2D (uT2D) was taken from the population with T2D in the study by Kontodinopoulos et al [28]. Men had a utility value of 0.724 (SE: 0.010) and women had a utility value of 0.701 (SE: 0.010).

The utility value of death was zero.

We used a 3% discount rate for both costs and QALYs [30]. We also performed sensitivity analyses for the discount rates of 0% and 5% and effectiveness assumptions of 3 years and 20 years.

Probabilistic modelling allows the joint effect of parameter uncertainty by probabilistic distribution of each or most input parameters [31]. Table 4 summarises the chosen statistical distributions for the model input parameters and provides additional background information.

**Table 4:** Chosen distributions for model input parameters

Parameter^1^	Deterministic value	Standard Error	Distribution	Alpha	Beta
**Ngt2igt**	0.163	0.037	Beta	16.28	83.72

**Igt2ngt**	0.162	0.037	Beta	16.23	83.77

**Igt2t2d**	0.062	0.024	Beta	6.23	93.77

**T2d2igt**	0.005	0.007	Beta	0.50	99.50

					

**cNGT**	EUR1,744.21.00	EUR1,744.21	Gamma	1.00	1744.21

**cIGT**	EUR2,696.48	EUR2,696.48	Gamma	1.00	2696.48

**cT2D**	EUR 5,896.48	EUR5,861.92	Gamma	1.00	5861.92

**Cost of intervention**	EUR230.03	EUR230.03	Gamma	1.00	230.03

					

	*Mean*		*Methods of moments*		

**uNGT, men**	0.772	0.004	Beta	7067.38	2087.26

**uNGT, women**	0.747	0.004	Beta	7342.67	2486.87

**uIGT, men**	0.764	0.006	Beta	3458.69	1066.73

**uIGT, women**	0.740	0.006	Beta	3578.37	1260.34

**uT2D, men**	0.724	0.010	Beta	1400.32	533.82

**uT2D, women**	0.701	0.010	Beta	1422.19	606.61

^1^ngt2igt: probability of moving from NGT to IGT state

igt2ngt: probability of moving from IGT to NGT state

igt2t2d: probability of moving from IGT to T2D state

t2d2igt: probability of moving from T2D to IGT state

cNGT: annual health care costs for NGT

cIGT: annual health care costs for IGT

cT2D: annual health care costs for T2D

uNGT: health utility value for NGT

uIGT: health utility value for IGT

uT2D: health utility value for T2D

## Results

The model can be adjusted to display results of men and women for different age groups. To facilitate presentation, six age-sex groups (ages 30, 50, 70 for men and women) are described. The results of all sub-groups for ICER, QALYs gained and costs are displayed in table 5.

**Table 5:** Results for incremental cost-effectiveness ratio (ICER), quality-adjusted life years (QALYs) gained and cost by age and sex groups

ICER					
Age	Sex				

	Men	Women			

30	-25,164 €	-31 407 €			

50	-15,108 €	-21,215 €			

70	27,546 €	19,433 €			

					

**QALYs gained**					

Age	Sex				

	Men	Women	Men, intervention	Women, intervention	

30	17.44	18.07	17.46	18.10	

50	13.24	14.06	13.27	14.08	

70	6.04	6.96	6.06	6.98	

					

**Cost**					

Age	Sex				

	Men	Women	Men, intervention	Women, intervention	

30	76 270 €	83,277 €	75,670 €	82,587 €	

50	52,652 €	59,236 €	52,308 €	58,787 €	

70	20,686 €	25,611 €	21,021 €	25,849 €	

For example, the ICER is negative for men aged 50 at the start of the intervention. The difference in costs and QALYs gained of no intervention vs. intervention for men aged 50 is EUR52,652 vs. EUR52,308 and 13.24 QALYs gained vs. 13.27 QALYs gained.

**Figure 2:**
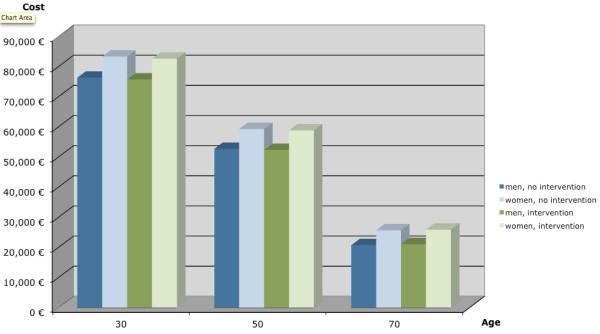
**Costs by age, sex and intervention**.

Figure 2 summarises the simulated costs of no intervention vs. intervention among women and men at different ages. Women have higher costs in all categories compared to men. The intervention leads to a cost-savings in comparison to no intervention for age groups 30 and 50. This means the intervention will save money in these age groups as fewer people will transition to higher-risk health states. For the group aged 70 years at the beginning of the intervention, the intervention group is more costly than the no-intervention group. For all categories, the costs decrease substantially with age at entry.

**Figure 3:**
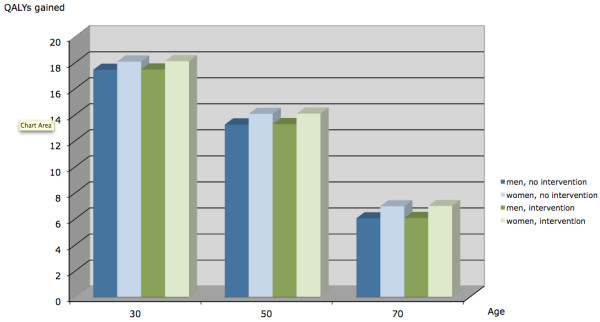
**Quality-adjusted life years (QALYs) gained by age, sex and intervention**.

Figure 3 compares the QALYs gained by sex and age groups for no intervention vs. intervention. Women have higher QALYs gained regardless of intervention group. Individuals in the intervention also had higher QALYs gained. The difference in QALYs gained between the intervention and no-intervention groups is small.

**Figure 4:**
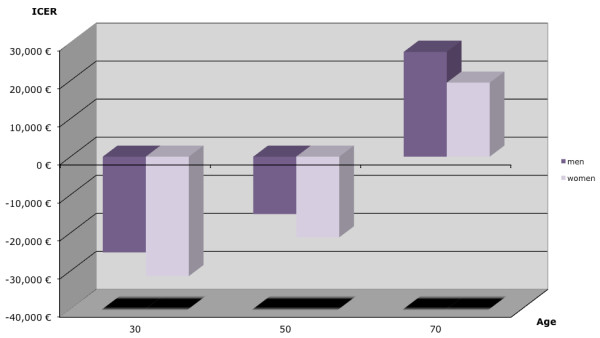
**Incremental cost-effectiveness ratios (ICERs) by age and sex**.

Figure 4 outlines the ICERs by age, sex and intervention. ICERs were negative for men and women aged 30 and 50 at the start of the intervention. ICERs were positive (men EUR27,546/QALY gained and women EUR19,433/QALY gained) for men and women aged 70 years. The older the individual is at cohort entry, the higher is the ICER. The ICERs are consistently lower in women.

**Figure 5:**
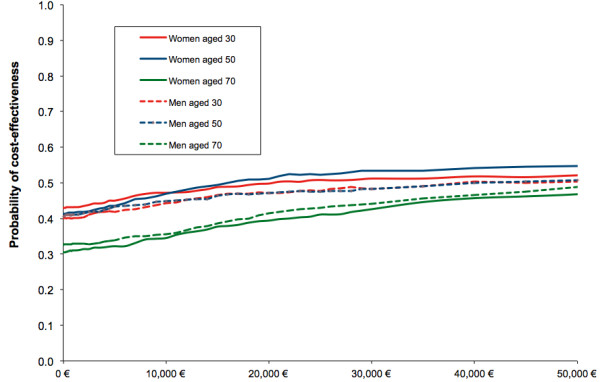
**Cost-Effectiveness Acceptability Curves for Different Age and Sex Characteristics**.

Figure 5 compares cost-effectiveness acceptability (CEA) curves by age and sex groups. The CEA curve illustrates the probability that the intervention is cost-effective given different willingness-to-pay threshold values [32]. The higher the selected threshold value, the higher the probability of the intervention being cost-effective. The curves range between a probability of cost-effectiveness of 30% and 55% (assumed willingness-to-pay threshold: EUR0 to EUR50,000). The slope of the curve is flat. Men and women in the same age group show similar CEA curves. Men and women in the older age group have a lower CEA curve. For an assumed willingness-to-pay threshold value of EUR50,000/QALY gained, the probability that the intervention is cost-effective is 45-55%. The threshold value of EUR50,000/QALY gained is arbitrary, but these graphs provide estimations of the development of the probability assuming different threshold values. The graph also allows visualisation of the uncertainty of the outcome. The presented CEA curves display an increasing probability of cost-effectiveness with increasing threshold value [17,32,33]. Nevertheless, the slope is unusually flat.

This model is a probabilistic model. Therefore, most input parameters are distributed according to a certain statistical distribution and consequently reflect uncertainty [31].

### Sensitivity analysis

A one-way sensitivity analysis was conducted for the discount rate and effectiveness of the intervention. Both changes have an impact on the results of the intervention. Changing the effectiveness showed a strong influence on the final result. The higher the assumed discount rate, the less effective the intervention. The greatest benefit of the intervention is in the future. If the duration of effectiveness is changed to 3 years, ie only effective for two years, interventions for all age and sex groups are not cost-effective if the assumed cost-effectiveness threshold is EUR50,000/QALY gained. Assuming 20-year effectiveness, all ICERs are negative and therefore cost-effective. See table 6 for an overview of sensitivity analyses for the discount rates of 0% and 5% and effectiveness assumptions of 3 years and 20 years.

**Table 6:** One-way sensitivity analyses of discount rate and intervention effectiveness assumption

ICER (deterministic) in Euros per QALY gained			
**Discount rate**			

	**0%**	**3%**	**5%**

**Man, 30 years**	-34,749	-25,164	-15,736

**Women, 30 years**	-43,831	-31,407	-20,212

**Man, 50 years**	-23,032	-15,108	-7,738

**Woman, 50 years**	-30,831	-21,215	-12,746

**Man, 70 years**	15,796	27,546	35,863

**Woman, 70 years**	5,935	19,443	29,074

**Effectiveness of intervention**			

	**3 years**	**7 years**	**20 years**

**Man, 30 years**	54,644	-25,164	-52,186

**Woman, 30 years**	55,441	-31,407	-61,140

**Man, 50 years**	65,552	-15,108	-39,813

**Woman, 50 years**	67,048	-21,215	-48,969

**Man, 70 years**	146,654	27,546	-13,294

**Woman, 70 years**	147,153	19,443	-24,960

In conclusion, the model indicates that diabetes prevention interventions have the potential to be cost-effective and even cost-saving, if older age groups are excluded. However, the uncertainty of the results is high. The sensitivity analysis shows that the model is sensitive to parameter changes.

## Discussion

### Principle findings

ICERs in the young and middle-age age groups (age 30 and 50) show cost-savings and are thus naturally below the EUR50,000/QALY gained that is routinely used as the threshold value for German health care interventions [34]. However, ICERs are positive for men and women aged 70 at the start of the intervention. This shows importance of the age at the intervention initiation. Even though all ICERs are considered cost-effective (below EUR50,000/QALY gained), interventions for men and women aged 30 and 50 specifically increase QALYs and decrease costs. ICERs for the younger age groups are thus not only cost-effective but also cost-saving. The CEA curves have a flat slope and indicate a probability that the intervention is cost-effective at less than or close to 50%. They are also uncertain results. In addition, the one-way sensitivity analyses for the discount rate and effectiveness reveal that the model is sensitive to changes of the programme effectiveness, even though most parameters were distributed probabilistically. We chose a conservative approach in selecting the duration of intervention effectiveness, and believe that the intervention could be even more cost-effective/cost-saving than suggested by the model.

The older the patient at the start of the intervention, the lower the costs and QALYs gained. These lower costs and QALYs gained are primarily due to older people being closer to death.

Compared to men, women have higher costs, higher QALYs gained and a larger savings per QALY gained in each age group. The higher QALYs gained occur because women have a greater benefit from prevention of diabetes. This is reflected in differences between utility values [28] in the different states. The higher costs arise because women live longer.

### Meaning of the study

This study provides results of a Markov model with cost per QALY gained as the final outcome using the latest evidence. The model merges the best available evidence but reveals shortcomings in data availability and parameter certainty. Further studies are needed to obtain these data. This study is the first to estimate the costs of a lifestyle intervention programme similar to the SDPP. The model underlines the importance of modelling all of the glycaemic states (ie, NGT and IGT) prior to diabetes rather than using only IGT. The cost-effectiveness acceptability curves can enhance decision-making capability by visualising uncertainty around the deterministic results.

### Strengths and Weaknesses of the Study

The study likely underestimates the health benefits that accrue to a diabetic patient since beneficial lifestyle changes will also reduce the incidence of other diseases. Considering the positive impact only on diabetes underestimates the positive influence on overall health. The evaluated lifestyle changes influence the risk of diabetes and diseases such as cardiovascular disease, cancer and other diseases and these health consequences are unaccounted for in the model. The same is true for production gains resulting from better health. Furthermore, screening (known to give valuable information from the individual's point of view) is also ignored in our analysis. To balance these shortcomings on the effect side, we excluded participant time costs.

The studies used for extraction of the different input parameters were based on different source populations, eg, Saxon, German, Finnish and US American. This limitation needs to be accepted because of the limited good evidence for use in modelling. We intended to do this analysis from a societal perspective, but available data made this only partially possible.

The DPS was conducted for a shorter time period than the current model. Thus, there are no reliable data on how motivation and compliance evolve over a longer period of time. On the other hand, the Chinese Da Qing Diabetes Prevention Study estimated that lifestyle intervention was effective at 20 years after the beginning of the intervention [22].

Besides the noted weaknesses, this study has numerous strengths. The CEA curves show considerable uncertainy when moving towards the most relevant willingness-to-pay threshold values. Considering just the deterministic ICER results shows that high cost-savings could hide the uncertainty of the results. The model is also transparent and can be adapted to local circumstances. Critical appraisal of the model is essential for future improvements. The study models the development of pre-diabetes and diabetes. This gives important insight into diabetes prevention and considers the possibility of conversion from diabetes to IGT. Furthermore, the analysis takes a conservative viewpoint. The participants were only able to participate in the intervention for five years. Thus, the costs only occur for five years. On the other hand, the effectiveness of the intervention is set to last only for seven years, with a linear decrease in effectivenes over the years. Furthermore, we assumed no difference in health utility between intervention and no intervention when they belonged to the same state. Studies have shown that lifestyle intervention participants have higher health utilities than those who do not participate [35,36]. Moreover, we assume that the probability of moving from diabetes is the same whether the person participates in the intervention or not.

Herman et al [36] evaluated the lifetime cost-utility of the DPP interventions in the United States and provided a higher ICER of US$1,100/QALY gained. Reasons for the higher ICER could be that the cost for the model started with an increased diabetes risk, ie IGT, the lifestyle intervention was higher, and it pertained to a lifelong duration. Caro et al [6] reported an ICER of CAS$749/life year gained. This result is not comparable as life years gained are not equivalent to QALYs gained. Daziel et al [7] estimated a cost per QALY gained of AU $1,880 [7]. However, the model was for 20 years. The benefits of early intervention could thus be underestimated. Gillies et al [8] calculated a cost of £6242 for screening for diabetes and impaired glucose tolerance followed by lifestyle interventions. The higher cost for one QALY gained could derive from a focus on screening and selection of individuals with at least one recognised risk factor for T2D for their primary model population [8]. Thus, the baseline population was considered to be at risk. Nevertheless, all the diabetes prevention models indicate that lifestyle intervention is cost-effective and this is consistent with our findings.

We found it difficult to compare utility weights from different studies. The different methods used to extract utility weights makes comparisons hard. In addition, no study on utility weights of Germans with normal glucose tolerance could be found.

We considered to externally validate the entire model but realised that, as mentioned above, not all necessary data a available yet. It can be possible in the future to "validate" the model against high quality cohort studies from Germany or Sweden or Spain, but such data does not exist today. We have however checked our model for consistency by including one-way and probabilistic sensitivity analyses. What we try to develop is a more "generic" model for some countries in Northern Europe, and we think that information based on a country mix is quite satisfaction for this purpose.

We considered to externally validate the entire model but realised that, as mentioned above, not all necessary data a available yet. It can be possible in the future to "validate" the model against high quality cohort studies from Germany or Sweden, but such data does not exist today. We have however checked our model for consistency by including one-way and probabilistic sensitivity analyses. What we try to develop is a more "generic" model for some countries in Northern Europe, and we think that information based on a country mix is quite satisfaction for this purpose.

### Unanswered Questions and Further Research

We encourage further research on utility weights of normal glucose tolerance, impaired glucose tolerance and type 2 diabetes mellitus. Multiple studies examine utility weights of diabetes and states of complications. However, a further emphasis should be placed on the states that precede diabetes and diabetes development. There is a profound lack of studies on the utilities of IGT and other prediabetic states.

As suggested by Roglic at al [25], the influence of hyperglycemia on mortality is greater than the impact of diabetes on mortality alone. Diabetes is a multi-faceted disease with a higher risk of developing other diseases such as heart disease and hypertension. Therefore, a comprehensive study on mortality due to diabetes is encouraged. We did not include the higher risk of dying due to hyperglycemia because the model results were inconsistent.

In addition, in order to draw more stable conclusions about the cost-effectiveness of lifestyle intervention programmes, more studies on the transition probabilities within interventions, programme resource use, and long-term effects and compliance would be very valuable.

These areas are encouraged for further research because they are the parameters that yield the greatest weaknesses in the model. Further investigation is needed to improve the model.

## Conclusions

Lifestyle interventions, as described in this article, are cost-saving for men and women aged 30 or 50 years at the start of the intervention and cost-effective for men and women aged 70 years. Nonetheless, there is a high degree of uncertainty around the ICERs. With the conservative approach adopted for this model, the long-term effectiveness of the intervention could be underestimated. After further evaluation of lifestyle interventions to prevent diabetes, this model should be updated with effectiveness data that comes directly from the intervention.

## List of abbreviations used

BMI: body mass index; FINDRISK: Finnish Type 2 Diabetes Risk Score; SDPP: Saxon Diabetes Prevention Programme; PREDIAS: Prevention of Diabetes Self-Management Program; ICER: incremental cost-effectiveness ratios; QALY: quality-adjusted life year; NGT: normal glucose tolerance; IGT: impaired glucose tolerance; T2D: type 2 diabetes mellitus; cNGT: annual health care costs for NGT; cIGT: annual health care costs for IGT; cT2D: annual health care costs for T2D; uNGT: health utility value for NGT; uIGT: health utility value for IGT; uT2D: health utility value for T2D; ngt2igt: probability of moving from NGT to IGT state; igt2ngt: probability of moving from IGT to NGT state; igt2igt: probability staying in IGT state; igt2t2d: probability of moving from IGT to T2D state; t2d2igt: probability of moving from T2D to IGT state; t2d2d: probability of dying from T2D state; mr[age]: probability of mortality in a certain age and sex group when T2D not present.

## Competing interests

The authors declare that they have no competing interests.

## Authors' contributions

AN drafted the concept and design of the study, created the model, searched for input parameters, coordinated the literature search, and was responsible for writing, analysis and revision. PS assisted in analysing the costs of the SDPP, and provided insight into the programme structure and expertise on diabetes prevention. LL provided health economic expertise, assisted in the model structure and economic evaluations, and supervised the writing, analysis and revision. All authors read and approved the final manuscript.
